# Is the risk of preeclampsia higher in donor oocyte pregnancies? A systematic review and meta-analysis

**DOI:** 10.5935/1518-0557.20180001

**Published:** 2018

**Authors:** Juan Enrique Schwarze, Paula Borda, Pamela Vásquez, Carolina Ortega, Sonia Villa, Javier A. Crosby, Ricardo Pommer

**Affiliations:** 1 Clinical Departament of Obstetrics and Gynecology, Universidad de Santiago, Chile; 2 Unit of Reproductive Medicine, Clínica Monteblanco, Santiago, Chile; 3 School of Dentistry, Universidad de los Andes, Santiago, Chile; 4 Unit of Reproductive Medicine, Clínica Las Condes, Santiago, Chile

**Keywords:** oocyte donation, preeclampsia, *in vitro* fertilization

## Abstract

**Objective:**

Preeclampsia (PE) occurs in 4.6% of pregnancies worldwide. The social phenomenon of increasing maternal age has raised the demand for donor oocytes. Egg donation has allowed women with poor ovarian reserve, premature ovarian failure, genetic disorders or surgical menopause to get pregnant. Recipients provide a unique model of immune response because of the differences in the genetic makeup of mothers and fetuses. In PE, immune tolerance may be impaired as a result of having non-autologous eggs implanted. Egg donation is a highly successful assisted reproductive technology, despite the significant number of issues arising from the implantation of non-autologous eggs. This study aimed to determine whether there is an association between egg donation and preeclampsia.

**Methods:**

A systematic review of the literature available in PubMed and Google Scholar was carried out from January of 1995 to August of 2016 using the terms 'oocyte donation, preeclampsia', 'oocyte donation, *in vitro* fertilization, preeclampsia', 'oocyte donation, preeclampsia, outcomes pregnancies', 'oocyte donation, obstetric outcome.' Only six retrospective cohort studies met the selection criteria.

**Result:**

The meta-analysis revealed a statistically significant association between egg donation and onset of preeclampsia (OR 4.50; 95% CI: 3.28-6.19; *p*<0.0001).

**Conclusion:**

Oocyte donation is associated with increased risk of preeclampsia in singleton pregnancies. Therefore, it is crucial to properly record and assess this finding when egg donation is the chosen assisted reproductive technology to attain pregnancy.

## INTRODUCTION

Estimates indicate that 4.6% of all pregnancies are complicated by pre-eclampsia (PE). Incidence varies depending on maternal age and on whether the mother is in her first pregnancy (Hutcheon *et al.*, 2011). Maternal age and the number of donor oocyte procedures have increased steadily in recent years (Berkowitz *et al.*, 1993; Ferraretti *et al.*, 2013).

Egg recipients provide a unique model of immune response because of the differences in the genetic makeup of mothers and fetuses (Levron *et al.*, 2014). The presence of an immune component in the etiology of preeclampsia has been suggested. While in pregnancies without PE there is a certain tolerance for foreign antigens, in the presence of preeclampsia immune tolerance is hampered in cases of non-autologous egg implantation (Redman & Sargent, 2010).

Many issues have been described in donor egg pregnancies (Van der Hoorn *et al.*, 2010). Previous studies have shown inconsistent results when comparisons were made between complications of *in-vitro* fertilization (IVF) procedures with and without donor oocytes, for reasons ranging from small simple sizes to inadequate control groups (Abdalla *et al.*, 1998; Krieg *et al.* 2008; Wiggins & Main 2005).

The purpose of this systematic review and meta-analysis was to determine whether donor oocyte pregnancies have higher incidences of preeclampsia when compared to IVF using autologous oocytes.

## MATERIAL AND METHODS

### Search

The search for literature was performed in MEDLINE via PubMed and Google Scholar (January 1995 to August 2016) using combinations of the terms "oocyte donation, preeclampsia", "oocyte donation, *in vitro* fertilization, preeclampsia", "oocyte donation, preeclampsia, outcomes pregnancies", "oocyte donation, obstetric outcome" in all search fields. Only papers written in English were included.

### Study selection

Cohort studies were eligible for inclusion in the review. Case reports, case series, and secondary studies were excluded. Studies comparing IVF, using donor eggs or autologous oocytes, were selected for inclusion. Women aged 35 to 49 years with singleton pregnancies and diagnosed with preeclampsia were eligible for inclusion. Studies enrolling patients with gestational hypertension and PE occurring concurrently were excluded.

### Data extraction

Two independent unmasked reviewers screened all potentially relevant papers for their titles and abstracts, and retrieved full texts only for the papers meeting the selection criteria. Disagreements were resolved with the involvement of a third reviewer (CO). The references of the selected papers were searched for additional studies. Data was extracted by one of the authors (PB) using a standardized extraction form that included number of cases with and without PE and *in vitro* fertilization pregnancies with or without donor eggs.

### Synthesis of studies

Studies were combined using a fixed-effect model. The statistical analysis included 2x2 contingency tables, from which ORs and their 95% confidence intervals were calculated for donor oocyte pregnancies using the Peto method. Meta-analysis was performed with Stata 11.0 (Statacorp, USA). Additionally, results were shown in a forest plot. Heterogeneity was evaluated using the I^2^ test and Cochrane's Q test. Heterogeneity was considered significant when *p*<0.1 and I^2^>40%.

## RESULTS

The search retrieved 193 papers. After manually excluding duplicates, 114 articles remained for screening by title and abstract. Only 18 studies remained for full text revision. Eight studies were excluded because their samples comprised women with multiple pregnancies (Simeone *et al.*, 2012; Klatsky *et al.*, 2010; Le Ray *et al.* 2012; Söderström-Anttila *et al.*, 1998; Tranquilli *et al.* 2013; Wiggins & Main 2005; Michalas *et al.*, 1996; Henne *et al.*, 2007); one was excluded due to undifferentiated diagnosis of PE with gestational hypertension (Jeve *et al.*, 2016); two did not record the number of singleton pregnancies with PE (Corradetti *et al.*, 2012; Krieg *et al.*, 2008); and one had a study group that was not homogeneous for oocyte donation (Porreco *et al.*, 2005). Additionally, references from the selected papers were checked for new unidentified studies. In the end, six papers meeting the inclusion criteria were considered in the meta-analysis (Nejdet *et al.*, 2016; Van Dorp *et al.*, 2014; Levron *et al.*, 2014; Malchau *et al.*, 2013; Stoop *et al.*, 2012; Salha *et al.*, 1999). The included papers featured retrospective cohort studies. The risk of PE was adjusted for maternal age and parity; in two studies, additional adjustments were made for fetal gender (Malchau *et al.*, 2013; Stoop *et al.*, 2012); and in one study adjustments were made for tobacco use and BMI (Nejdet *et al.*, 2016).

The six selected studies evinced an association between donor oocytes and preeclampsia (PE) (37,994 pregnancies with 111 donor oocytes). Nejdet *et al.* (2016) included cases from 2003 to 2012 from *in vitro* fertilization clinics. In the donor oocyte group, 13.14% (51/338) had PE, whereas in the control group 4.14% (1.105/26.696) presented PE (OR 9.059; 95% confidence interval (CI): 5.518-14.874). Van Dorp *et al.* (2014) included cases from the Dutch Perinatal Register between 1992 and 2009. In the donor oocyte group, 13.11% (8/61) presented PE, while 10.94% (21/192) presented PE in the control group (OR 1.238; 95% CI: 0.502-3.053). Levron *et al.* (2014) included cases from 2005 to 2011. In the donor oocyte group, 9.35% (13/139) showed PE, while in the control group 4.76% (6/126) had PE (OR 1.99; 95% CI: 0.782-5.052). Malchau *et al.* (2013) studied cases from 1995 to 2010 from the Danish IVF register. In the donor oocyte group, 9.76% (21/215) had PE whereas in the control group 3.21% (316/9.833) had PE (OR 7.55; 95% CI: 3.565-15.993). Stoop *et al.* (2012) analyzed cases from the Brussels center for reproductive medicine between 1999 and 2008. In the donor oocyte group, 15 of 148 patients (10.13%) had preeclampsia (OR 1.930; 95% CI: 0.825- 4.515). Salha *et al.* (1999) investigated cases at the St. James's University Hospital in Leeds. In the donor oocyte group, 13.63% (3/22) had PE, versus 3.8% (1/26) in the control group (OR 3.51; 95% CI: 0.459 -26.781) (Table 1).

**Table 1:** Characteristics of included studies

Author, year	Methods	Participants, period	Findings
*Nejdet, 2016*	Retrospective cohort study	Women from IVF Swedish clinics, from 2003 to 2012	Donor oocyte group consisted of 388 patients, 51 with PE (13.14%). In the control group, 1,105/26,696 (4.14%) had PE. OR 9.059 (95% CI:5.518 to 14.874). Weight: 41.31.
*Van Dorp, 2014*	Retrospective cohort study	Women from the Dutch Perinatal Register, from 1992 to 2009	Study group consisted of 61 patients with singleton pregnancies. 8 out of 61 had PE (13.11%). The control group had 21/192 (10.94%) cases of PE. OR 1.238 (95% CI:0.502 to 3.053). Weight 12.47.
*Levron, 2014*	Retrospective cohort study	From 2005 to 2011.	13 of 139 patients in the donor oocyte group had PE (9.35%). In the autologous oocyte group, 6 of 126 had PE (4.76%). OR 1.988 (95% CI:0.782 to 5.052). Weight 11.67.
*Malchau, 2013*	Retrospective cohort study	Women from the Danish *in vitro* fertilization register, from 1995 to 2010.	The donor oocyte group included 215 patients and 21 cases of PE (9.76%). Control group had 316/9.833 (3.21%) with PE. OR 7.551 (95% CI:3.565 to 15.993). Weight 18.03.
*Stoop, 2012*	Retrospective cohort study	Matched pair-analysis for age, ethnicity, parity and plurality from the Centre for Reproductive Medicine,Brussels. Data obtained from 1999 to 2008.	Donor oocyte had 15 of 148 pregnant women diagnosed with PE (10.13%). Control group had 8 of 148 (5.4%) with PE. OR 1.930 (95% CI:0.825 to 4.515). Weight 14.07.
*Salha, 1999*	Retrospective cohort study	Women from St. James's University Hospital, Leeds(UK), from 1992 to 1997	Donor oocyte group had 3 of 22 patients diagnosed with PE (13.63%). In the control group, only 1 pregnant woman of 26 was diagnosed with PE (3.8%). OR 3.508 (95% CI:0.459 to 26.781). Weight 2.46.

**Figure 1:**
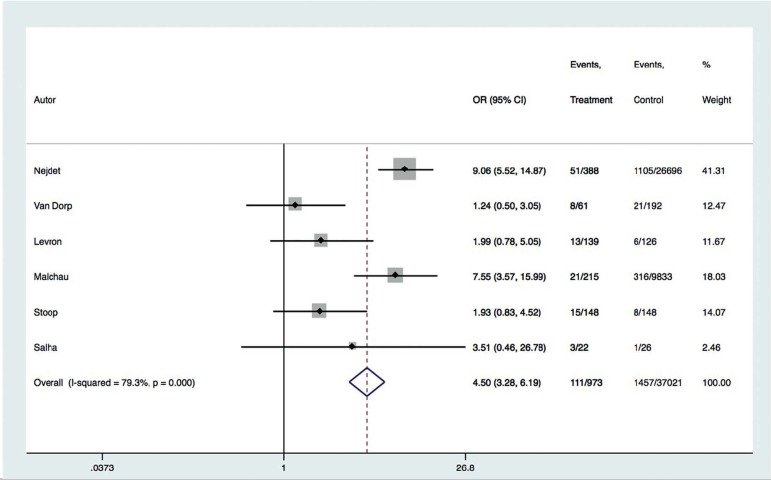
Forest plot

Preeclampsia was present in 11.5% of the pregnant patients with donor oocytes (n=111), versus 3.9% (n=1,457) of the individuals with autologous oocytes. After the meta-analysis, women who conceived using donor oocytes showed a significant increment in the risk of preeclampsia (RR 2.62; 95% CI: 2.13-3.21) (Figure 1). In general, heterogeneity across studies was assessed as not relevant (*p*=0.175; I2=34.9%).

**Figure 2:**
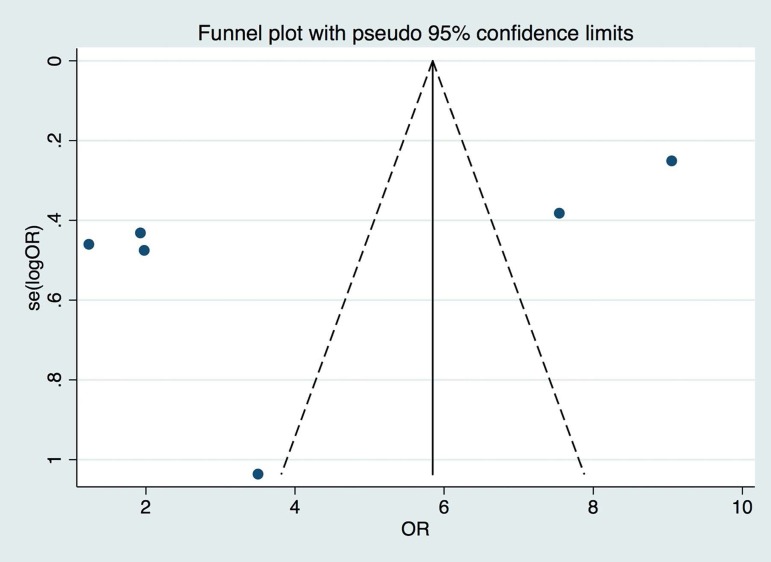
Funnel Plot

Publication bias was assessed using a funnel plot (Figure 2), and significant scatter of effect size and study size was found in both directions.

## DISCUSSION

The purpose of this systematic review and meta-analysis was to determine whether donor oocyte pregnancies were associated with increased risk of preeclampsia. In this review, a summary of six articles comparing the frequency of preeclampsia according to oocyte origin - autologous or donor - was performed. The findings revealed a significant increase in preeclampsia when donor oocytes were used.

A major strength of this study was that it focused exclusively on studies that compared IVF patients with oocyte origin as the variable of interest. Another important feature to be considered is that only singleton pregnancies were incorporated; maternal age and parity were accounted for in the analysis. A study limitation is the lack of information on the underlying causes for choosing of IVF with or without donor eggs, since the etiology of infertility might have been an independent risk factor for preeclampsia. All included papers featured retrospective cohort studies, which lack methodological robustness.

The etiology of preeclampsia in donor oocyte pregnancies is yet to be clarified. An immune theory has been postulated based on the allogenicity of the fetus in relation to the mother. In the implantation phase of pregnancy, the uterine decidua is invaded by trophoblast cells expressing HLA-C, a ligand of the immunoglobulin-type receptor of natural killer (NK) cells. NK cells facilitate the neovascularization of the decidua through proangiogenic and endothelial factors, which in turn modulate the adaptive changes of the uterine spiral arteries (Blázquez *et al.*, 2016). When this process unfolds appropriately, adequate blood flow to the fetus is guaranteed. Fetal HLA-C differs from maternal HLA-C because it contains paternal alleles. When a donor egg is used, fetal HLA-C is even less recognizable by the maternal immune system, as it is completely allogeneic. This can disturb the blood flow to the placenta and, in turn, facilitate the onset of certain disorders such as preeclampsia and intrauterine growth restriction (Klatsky *et al.*, 2010; Madeja *et al.*, 2011; Hiby *et al.*, 2004). The role of acetylsalicylic acid in preeclampsia prevention in women given donor oocytes is yet to be elucidated.

The evidence summarized in this paper revealed an association between oocyte origin and incidence of preeclampsia in singleton pregnancies from IVF. We believe that this study will allow physicians and patients to make informed decisions about the fertilization procedures available needed to attain reproductive success.
